# Identification of Markers Associated with Yield Traits and Morphological Features in Maize (*Zea mays* L.)

**DOI:** 10.3390/plants8090330

**Published:** 2019-09-05

**Authors:** Agnieszka Tomkowiak, Jan Bocianowski, Łukasz Wolko, Józef Adamczyk, Sylwia Mikołajczyk, Przemysław Łukasz Kowalczewski

**Affiliations:** 1 Department of Genetics and Plant Breeding, Poznań University of Life Sciences, 11 Dojazd St., 60-632 Poznań, Poland; 2 Department of Mathematical and Statistical Methods, Poznań University of Life Sciences, 28 Wojska Polskiego St., 60-637 Poznań, Poland; 3 Department of Biochemistry and Biotechnology, Poznań University of Life Sciences, 11 Dojazd St., 60-632 Poznań, Poland; 4 Plant Breeding Smolice Ltd., Co., Smolice 146, 63-740 Kobylin, Poland; 5 Institute of Food Technology of Plant Origin, Poznań University of Life Sciences, 31 Wojska Polskiego St., 60-624 Poznań, Poland

**Keywords:** genome-wide association mapping, candidate gene association mapping, DArTSeq, SNP

## Abstract

Association mapping is a powerful approach to detect associations between traits of interest and genetic markers based on linkage disequilibrium in molecular plant breeding. The aim of this study was the identification of single nucleotide polymorphisms (SNPs) and SilicoDArT markers associated with yield traits and morphological features in maize. Plant material constituted inbred lines. The field experiment with inbred lines was established on 10 m^2^ plots in a set of complete random blocks in three replicates. We observed 22 quantitative traits. Association mapping was performed in this study using a method based on the mixed linear model with the population structure estimated by eigenanalysis (principal component analysis applied to all markers) and modeled by random effects. As a result of mapping, 969 markers (346 SNPs and 623 SilocoDArT) were selected from 49,911 identified polymorphic molecular markers, which were significantly associated with the analyzed morphological features and yield structure traits. Markers associated with five or six traits were selected during further analyses, including SilicoDArT 4591115 (anthocyanin coloration of anthers, length of main axis above the highest lateral branch, cob length, number of grains per cob, weight of fresh grains per cob and weight of fresh grains per cob at 15% moisture), SilicoDArT 7059939 (anthocyanin coloration of glumes of cob, time of anthesis—50% of flowering plants, time of silk emergence—50% of flowering plants, anthocyanin coloration of anthers and cob diameter), SilicoDArT 5587991 (anthocyanin coloration of glumes of cob, time of anthesis—50% of flowering plants, anthocyanin coloration of anthers, curvature of lateral branches and number of rows of grain). The two genetic similarity dendrograms between the inbred lines were constructed based on all significant SNPs and SilicoDArT markers. On both dendrograms lines clustered according to the kernel structure (flint, dent) and origin. The selected markers may be useful in predicting hybrid formulas in a heterosis culture. The present study demonstrated that molecular SNP and Silico DArT markers could be used in this species to group lines in terms of origin and lines with incomplete origin data. They can also be useful in maize in predicting the hybrid formula and can find applications in the selection of parental components for heterosis crossings.

## 1. Introduction

Modern maize breeding is to create new cultivars with improved traits [1]. Currently, the breeding based largely on the use of the heterosis phenomenon (F1 hybrid vigor), occurring as a result of crossing two inbred lines with the highest combining ability. As a result, high-yielding hybrids are obtained with traits that exceed parental lines. Although the reasons for the occurrence of heterosis are not clearly determined, there are many hypotheses explaining this phenomenon, e.g., the hypothesis of overdomination or domination. It is believed that heterosis is associated with the genetic distance between parental forms, determined by DNA polymorphism, therefore adequate selection of parental components becomes a key element in the breeding process [1]. Presently, intensive research have been conducted on the possible use of molecular markers in the selection of parental lines for heterosis crosses [2,3,4]. Markers based on single nucleotide polymorphisms (SNPs) are increasingly used for this purpose [5].

Modern methods for identifying single SNP polymorphisms use the next-generation sequencing technology (NGS). The NGS is a technique developed in the 21st century that provides much higher performance and throughput than the previously used Sanger sequencing technique [6]. This technology provides inexpensive whole genome sequence readings through methods, such as chromatin immunoprecipitation, mutation mapping, polymorphism detection and detection of non-coding RNA sequences [7]. Sequencing methods such as: Restriction site associated DNA (RADseq) [8], multiplexed shotgun genotyping (MSG) [9] and bulked segregant RNA-Seq (BSRSEq) [10] enable the identification of a significant number of markers and more accurate examination of many loci in a small number of samples.

Another genotyping-by-sequencing method applied already to many hundreds of organisms is DArTseq™. The DArTseq™ represents a combination of a DArT complexity reduction methods and next generation sequencing platforms [11,12,13,14,15]. Therefore, DArTseq™ represents a new implementation of sequencing of complexity reduced representations [16] and more recent applications of this concept on the next generation sequencing platforms [8,17]. The DArTseq procedure [15] is used, among others, to identify single nucleotide markers (SNPs) and provides a large pool of so-called silicoDArTs that have a dominant character because the variability is determined by a single point mutation, without a variant from the second homologous chromosome. The presence or absence of a mutation (silicoDArT marker) is often treated as a single feature, which is assigned a value of 1 or 0, respectively. In this method, the genome complexity is reduced by restriction enzyme digestion and sequencing of short fragments [12]. DArTseq technology replaces the hybridization stage with sequencing taking place in the Illumin system [18]. Similar to DArT methods based on array hybridizations, the DArTseq™ technology is optimized for each organism by application the most appropriate complexity reduction method (both the size of the representation and the fraction of a genome selected for assays). DArTseq™ has been optimized for maize a few years ago and was used to characterize a complete maize germplasm collection of the International Maize and Wheat Improvement Center (CIMMYT).

Association mapping also called linkage disequilibrium mapping involves searching for genotype–phenotype correlations in unrelated individuals using dedicated statistical methods [19,20,21]. The association mapping approach provides possibilities to generate good quality markers for marker-assisted selection (MAS). Functional markers tightly linked with the trait reflect gene polymorphisms, which directly cause phenotypic variation. Association mapping provides opportunities to find such markers in a broad spectrum of genetic resources. Its potential results from the likelihood of higher mapping resolution, due to the use of a larger number of recombination events in the germplasm’s developmental history [22]. Thus, association mapping has become a promising approach compared to traditional mapping. There are two main types of association mapping: Genome-wide association mapping (GWAM) and candidate gene association mapping (CGAM). The GWAM approach surveys genetic variation in the whole genome to find association signals for various complex traits, whereas CGAM correlates DNA polymorphisms in selected candidate genes and the trait of interest [20,21]. There are many examples of successful application of association analysis in cereals, mainly in maize.

Recently, GWAM has evolved as a powerful tool to dissect the genetic architecture of complex traits in crop species [22,23]. Advances in NGS allow identification of thousands of genetic marker loci, which in turn enables their statistical association with traits of interest based on linkage disequilibrium [24]. Skim-based genotyping by sequencing (skimGBS) uses low-coverage (1–10×) whole genome sequencing for high resolution genotyping. Genomic reads from parental individuals are mapped to the reference genome and SNPs are predicted. Reads from the progeny are then mapped to the same reference and comparison with the parental SNP file enables calling of SNPs in the progeny of one or other parental genotypes [25]. Associated genetic markers can be causal for the trait of interest or in linkage disequilibrium with a causal locus [20]. To date, GWAM approaches using whole genome sequencing have allowed researchers to dissect genetic regulation of complex traits, such as oil biosynthesis, carotenoid concentration and yield in well studied crops, including maize and rice [26,27,28].

The aim of this study was to identify SNP and SilicoDArT markers associated with yield traits and morphological features in maize (*Zea mays* L.). These studies are to facilitate the selection of parental components for heterosis crossings.

## 2. Results

### 2.1. Phenotyping

**Figure 1:**
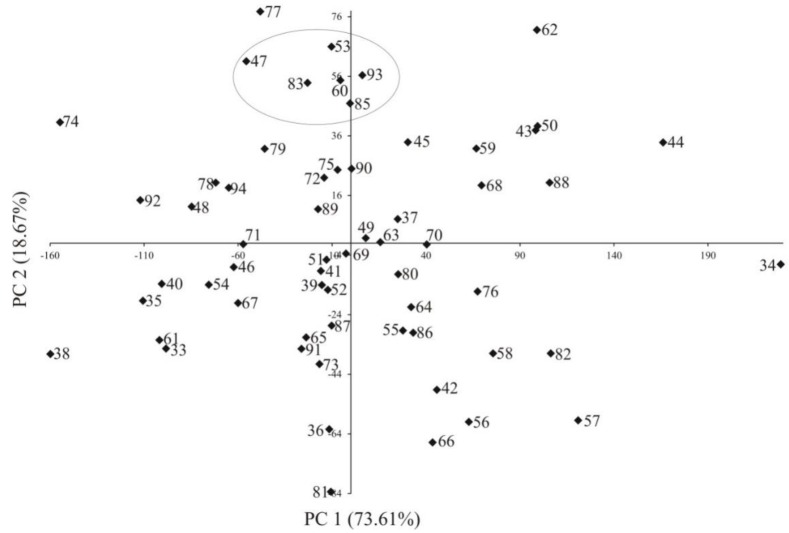
Population structure of inbred lines of maize (*Zea mays* L.) estimated by eigenanalysis. Flint F2: 74; Flint F2/EP1: 35,39,41,51,53; Flint F2/CM7: 73; Flint/BSSS: 38; Flint/ID: 40; Flint/Lancaster: 60,68; German Flint/F2: 69,82,93; Flint Origin unknown: 42,52,36; Dent ID: 33,43,44,56,58,59,61,62,64,65,72,76,79,80,83,85,88,89,90,94; Dent BSSS: 47,49,50,87; Dent ID/BSSS: 54,57,63,66,67,75,77,78,81,84,86,91,92,45,46; Dent Lancaster: 55; Dent ID/Lancaster: 50; Semident BSSS: 71.

The results of ANOVA demonstrated that the differences between lines were significant for all traits. Population structure estimated by eigenanalysis showed that lines formed randomly distributed groups (Figure 1). The generated groups consisted of lines with flint and dent grain structure of different origins, e.g., one of the groups consisted of lines: L47, L53, L60, L83, L85 and L93, which belonged to different origin groups. 

Table 1 shows correlation coefficients between the observed traits. The 54 pairs of traits were statistically significantly correlated positively, however 34 pairs correlated negatively.

### 2.2. Genotyping Data (SilicoDArT and SNP)

**Figure 2:**
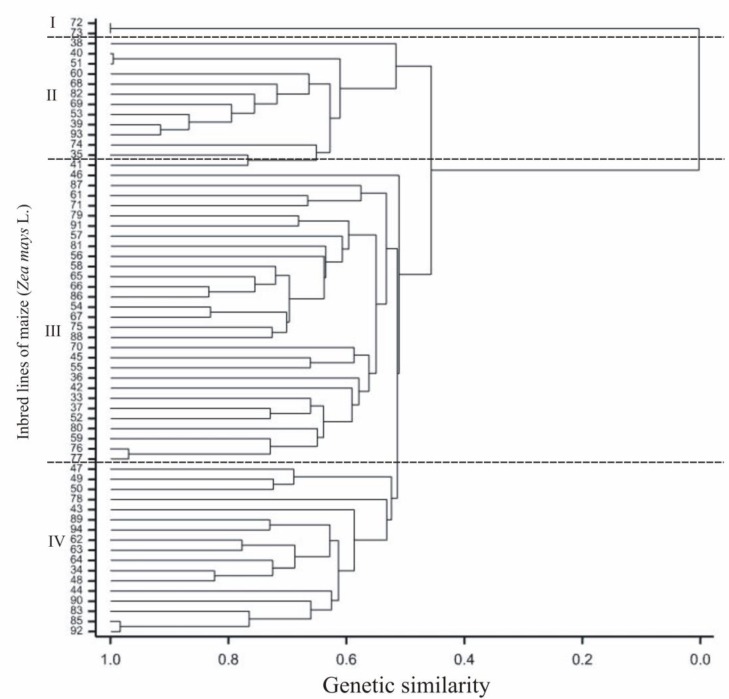
Dendrogram of genetic similarity of the studied inbred lines of maize (*Zea mays* L.) on the basis of single nucleotide polymorphism (SNP) marker observations.

**Figure 3:**
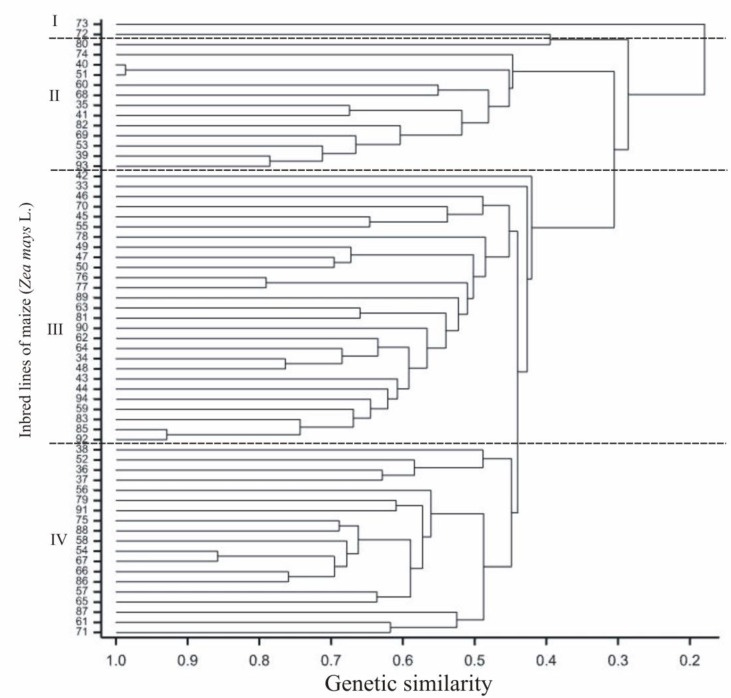
Dendrogram of genetic similarity of the studied inbred lines of maize (*Zea mays* L.) on the basis of SilicoDArT marker observations.

The DArTseq NGS analysis of tested maize lines allowed us to identify 49,911 polymorphisms (including 33,452 SilicoDArT and 16,459 SNP). Out of these, 3229 of these markers (including 2121 SilicoDArT and 1108 SNPs) were selected for GWAM using the following criteria: One SilicoDArT and SNP within a given sequence (69 nt), minor allele frequency (MAF) >0.25 and the missing observation fractions <10%. The two independent dendrograms (UPGMA) based on the SNP and SilicoDArT markers (calculated according to the Nei and Li’s distance [29] show the genetic relationships of 62 used inbred lines (Figure 2 and Figure 3)). The highest genetic similarity, calculated on the basis both types of markers (equal to 0.99) was revealed between the L40 and L51 lines, whereas the lowest genetic similarity (equal 0.062) was found for L53 and L72. Genetic similarity coefficients calculated based on the observations of markers of particular types and all markers together were significantly statistically correlated at the level of 0.001: Between SilicoDArT and SNPs *r* = 0.76, between SilicoDArT and all markers *r* = 0.95 and between SNPs and all markers *r* = 0.93.

Based on SNP markers the lines could be classified into three groups. The first group included the L72 line with the dent-type grain belonging to the Iowa Dent origin group and the L73 line with the flint grain originating from France. The second group consisted of all lines with the flint-type grain, where the L40 and L51 lines originating in Europe showed the highest similarity (98%). Parental forms of the L40 line were derived from France and Spain, while L51 from Germany. In the same group, similarity at the level of 90% occurred between the L39 and L93 lines; both of these lines were related to the F2 line bred at Institute National de la Recherche Agronomique (INRA) in France. The L38 line, which is the only one derived from the cross of a flint grain line with a line belonging to the Iowa Stiff Stalk Synthetic origin group from the USA, was the least related (52%) with other lines in this group. The similarity determined on the basis of SNP markers between the flint-type grain lines in the second group ranged from 52% to 98%. The third group included all dent- and semident-type grain lines with the exception of three flint-type grain lines, L42, L52 and L36 (all of unknown origin), which belonged to the same subgroup. The third group contained lines from the United States and the similarity between them ranged from 53% to 97% (between the L85 and L92 lines). The L85 line belonged to the Iowa Dent origin group, while the parental forms of the L92 line were from the Iowa Dent and Iowa Stiff Stalk Synthetic origin groups. The grouping of lines based on SNP marker similarity largely reflected the origin of these lines and the division based on the kernel structure (flint, dent; Figure 2).

Four basic groups could be distinguished between the analyzed lines on the similarity dendrogram of SilicoDArT markers. In the first group, similarly to the SNP-based tree, there was the L73 flint-type grain line. The second group contained the L72 and L80 dent-type grain lines, both from the Iowa Dent origin, and the similarity between them was 40%. The third group included all flint-type grain lines, where the highest similarity (95%) occurred between the L40 and L51 lines originating in Europe, similarly as in SNP markers. The similarity at the level of 78% was found between the L39 and L93 lines in the same group, similarly as in SNP markers. The fourth group included all dent- and semident-type grain lines, with the exception of four flint-type grain lines, L36, L42, L52 (all of unknown origin) and L38 (derived from the crossing of a flint-type grain with a dent-type grain form). Maize lines also clustered according to the kernel structure (flint, dent) and origin (Figure 3) in the case of Silico DArT markers, as in the similarity estimated on the basis of SNP markers.

**Figure 4:**
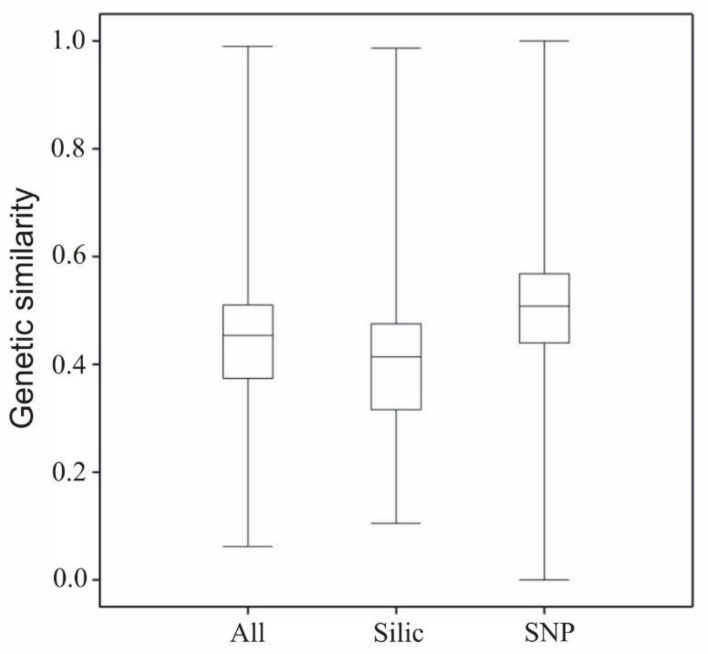
Boxplot of genetic similarity in two types of markers (SNP and SilicoDArT) and both types combined.

The variability of genetic similarity of studied lines calculated on the basis of SNP and SilicoDArT markers was not significantly different, as shown in the boxplot (Figure 4). The bottom and top of the box were the 25th and 75th percentiles—the lower and upper quartiles—, respectively, and the band near the middle of the box was the 50th percentile—the median. The ends of the whiskers represent the minimum and maximum of all data.

### 2.3. Association Mapping

The number of markers significantly associated with investigated traits at false discovery rate (FDR) < 0.05 was 969 in GWAM: 623 of SilicoDArTs and 346 of SNPs (Table 2). The least markers (6) were associated with the number of primary lateral branches (NPLB) trait, and the most (150) with the anthocyanin coloration of glumes of cob (ACGC) trait. Table S1 presents markers associated with at least two traits, i.e., characterized by pleiotropy—a very desirable trait in breeding. Markers associated with five or six traits deserve particular attention: SilicoDArT 4591115 (ACA, LMA, LC, NGC, WFGC and WFG15), SilicoDArT 7059939 (ACGC, TA, TSE, ACA and DC) and SilicoDArT 5587991 (ACGC, TA, ACA, CLB and NRG).

The sequences (69 bp length) of SilicoDArTs 4591115, 7059939 and 5587991 were used for physical mapping. These three markers were selected because they were associated with five or six traits. The BLAST search in the *Zea mays* reference genome enabled identification of the position of markers. The marker 4591115 was aligned with chromosome 5 in the non-coding region. The closest genes were distanced by 60 Kb at the 5′ side putative MYB DNA-binding domain superfamily protein (GLK47, XM_008683029) and vegetative cell wall protein gp1-like (LOC103628816, XM_008648941). At the 3′ side, 10 Kb away was the localized uncharacterized protein LOC100191236 (NM_001136670.1).

The Blast search was not able to identify any places in the genome fully identical to the sequences of SilicoDArT markers 5587991 and 7059939. The fragment (41 bp) of the marker 5587991 showed homology to the chromosome 1, in the region that was 78 Kb away from the myosin-1 gene. The different fragment of this marker (28 bp) was aligned to chromosome 3 within the intron of the 50S ribosomal protein L31 gene.

The two fragments (27 and 22 bp) of marker 7059939 were aligned to the separate regions of chromosome 2 (120 Mb distant from each other). The first fragment was located within the serine carboxypeptidase-like gene and the second was in protein LOC103648845. Another small fragment (25 bp) of this sequence showed homology to non-coding regions of chromosomes 4 and 9.

## 3. Discussion

Maize, one of our most important crop species, has been the target of genetic investigation and experimentation for more than 100 years. Crossing two inbred lines tends to result in “better” offspring, a process known as heterosis. Attempts to map genetic loci that control traits important for farming have been made, but few have been successful [30].

The DArTseq technology is a modification of the DArT method. It consists in replacing the hybridization step on microarrays with next-generation sequencing in the Illumina system [18]. Several times more polymorphic markers—both dominant silicoDArT and codominant SNPs—are obtained as a result of the analysis.

The first association mapping (AM) was described in wheat [31], where markers associated with resistance to cereal rust, yellow rust, powdery mildew and also grain yield were identified. One hundred and seventy winter wheat (*Triticum aestivum* L.) lines were analyzed. A number of markers of the traits studied was selected based on AM, and they were positioned on the appropriate species chromosomes, based on the genetic map containing 1644 markers, of which 813 were DArT markers. DArT markers have also proved useful in association mapping in wheat [32]. The latter authors identified markers highly associated with important agrotechnical traits useful in breeding programs. DArT markers have been successfully used to analyze the genetic diversity and structure of Chinese common wheat (*Triticum aestivum* L.) populations. A total of 111 cultivars and breeding lines from northern China were examined. The results provided information for further selection of parental forms and establishing heterozygous test materials for the needs of the Chinese wheat breeding program [33].

In the present study, similarity dendrograms were constructed between the inbred lines based on all significant SNPs and SilicoDArT markers. Inbred lines on both dendrograms clustered according to the kernel structure (flint, dent) and origin. There were no statistically significant differences in the clustering of the analyzed lines between genetic similarity results based on SNP and SilicoDArT markers.

The association mapping resulted in identification of 969 markers significantly linked (at FDR < 0.05 in GWAM) with the analyzed morphological features and yield structure traits. Among the selected markers, 623 were SilicoDArTs and 346 were SNPs. The least markers (6) were associated with the NPLB trait, and the most (150) with the anthocyanin coloration of glumes of cob. Three markers were associated with five or six traits: SilicoDArT 4591115 (anthocyanin coloration of anthers, length of main axis above the highest lateral branch, cob length, number of grains per cob, weight of fresh grains per cob and weight of fresh grains per cob at 15% moisture), SilicoDArT 7059939 (anthocyanin coloration of glumes of cob, time of anthesis—50% of flowering plants, time of silk emergence—50% of flowering plants, anthocyanin coloration of anthers and cob diameter) and SilicoDArT 5587991 (anthocyanin coloration of glumes of cob, time of anthesis, anthocyanin coloration of anthers, curvature of lateral branches and number of rows of grain). The sequence of SilicoDArTs 4591115, 7059939 and 5587991 were used in physical mapping in the *Zea mays* genome. The marker 4591115 was localized on chromosome 5 on the non-coding region. In the closest neighborhood (10–60 Kb) were localized putative MYB DNA-binding domain protein, vegetative cell wall protein gp1-like gene and some uncharacterized protein LOC100191236. It is difficult to conclude whether the localization of this marker in the genome can have a direct meaning on its detected correlations with the features.

One fragment (41 bp) of SilicoDArT 5587991 showed homology to chromosome 1, and the second fragment of this marker (28 bp) was aligned to chromosome 3 within the intron of the 50S ribosomal protein L31 gene. The two fragments (27 and 22 bp) of marker 7059939 were aligned to the separate regions of chromosome 2 (120 Mb distant from each other). The first fragment was located directly within the serine carboxypeptidase-like gene and the second was in protein LOC103648845. Both markers showed homology to the coding sequences, which might affect the correlated features. It is difficult to explain division of the marker sequences into two fragments. It could be the result of incidental ligation during the library preparation step or alternatively real rearrangements in tested lines genomes.

The theoretical basis for the relationship between genetic distance and heterosis was presented by Bernardo [34]. He found that molecular markers could be useful for predicting heterosis if they show a strong domination effect, allele frequency is negatively correlated with parents, their inheritance is high and there is an association between quantitative trait loci (QTL).

In the study, similarity dendrograms were created between inbred lines based on molecular markers. The lines analyzed clustered according to origin. Most studies indicate that the less related parental components, the higher the heterosis effect can be expected in the F1 generation hybrids. Thus, in the case of missing or incomplete information about the origin of parental lines, molecular SNP and SilicoDArT markers may be useful in predicting hybrid formulas in heterosis.

Nineteen tropical maize biparental populations evaluated in multienvironment trials were used in Zhang et al.’s [35] study to assess the prediction accuracy of different quantitative traits using low-density (~200 markers) and genotyping-by-sequencing (GBS) single-nucleotide polymorphisms (SNPs), respectively. An extension of the genomic best linear unbiased predictor that incorporates genotype × environment (GE) interaction was used to predict genotypic values; cross-validation methods were applied to quantify prediction accuracy. Their results showed that low-density SNPs were largely sufficient to obtain a good prediction in biparental maize populations for simple traits with moderate-to-high heritability, but GBS outperformed low-density SNPs for complex traits. GE interaction in maize is usually strong for complex quantitative traits, and maize hybrids are always tested in multiple environments. Most of the current genomic prediction studies have only applied a single-environment model and have not considered predictive models with correlated environmental structures [36]. Cook et al. [37] conducted joint-linkage quantitative trait locus (QTL) mapping and GWAM for kernel starch, protein and oil in the maize nested association mapping population, composed of 25 recombinant inbred line families derived from diverse inbred lines. Joint-linkage mapping revealed that the genetic architecture of kernel composition traits is controlled by 21–26 QTLs. Numerous GWAM associations were detected, including several oil and starch associations in acyl-CoA:diacylglycerol acyltransferase 1–2, a gene that regulates oil composition and quantity. Results from nested association mapping were verified in a 282 inbred association panel using both GWAM and candidate gene association approaches. They identified many beneficial alleles that will be useful for improving kernel starch, protein and oil content. Benke et al. [38] investigated the effect of two different Fe regimes on the formation of morphological and physiological traits; they have identified polymorphisms significantly associated with morphological and physiological traits and analyzed the correlation between those traits employing the association mapping population. Fine mapping of QTL confidence intervals of the intercrossed B73 × Mo17 population resulted in the identification of a total of 13 SNPs in Fe limited regime and 2 SNPs under normal supplementation that were statistically (FDR = 0.05) associated with cytochrome P450 94A1, invertase beta-fructofuranosidase insoluble isozyme 6 and a low-temperature-induced 65 kDa protein. Association analysis of the entire genome under restricted and normal Fe treatments yielded a total of 18 and 17 significant SNPs, respectively. Dell’Acqua et al. [39] generated for the first time a balanced multi-parental population in maize, which serves as a tool for effortless QTL mapping in maize due to a large variety and dense recombination events. This author generated 1636 MAGIC maize recombinant inbred lines originating from eight genetically different founder lines. The analysis of the MAGIC 529 maize line demonstrated that the population is a balanced, uniformly differentiated mosaic of eight founders that has a mapping power and resolution enhanced by the high frequencies of minor alleles and the rapid disappearance of linkage disequilibrium. That study provided evidence how MAGIC maize can be used to find strong candidate genes through the incorporation of genome sequencing and transcriptomic information. The latter authors described three flowering time QTLs and three grain yield QTLs and indicated potential candidate genes. MAGIC maize subsets have been demonstrated to acquire high power and high resolution QTL mapping in power simulations. According to Xiao et al. [40], a growing number of readily available GWAM results allow us to narrow down association analyses to single well-annotated candidate genes and to elucidate the structure of the genome and its constitution connected with the studied traits. First attempts aimed at calculating the pattern of the distribution of associated loci at the whole genome level have demonstrated that intragenic regions and those with close proximity to genes (as opposed to intergenic regions) were primarily responsible for the variability of maize traits, particularly in the 5′UTR (non-translated region) [10]. In addition, non-synonymous mutated SNPs along with variants with high copy numbers show the highest rate of functional mutations, while intergenic regions contain significantly less functional SNPs [41]. The above systematic studies indicate that gene regulation at the level of expression should have an important function in phenotypic diversity. The expression pattern of immature maize kernels has been broadly studied within the frame of this hypothesis [42] and highly similar conclusions have been drawn as in earlier studies regarding quantitative traits; namely that non-synonymous SNPs are the crucial factors in expression regulation, and they have the highest number of SNP-QTL associations [42].

## 4. Materials and Methods

### 4.1. Plant Materials

The plant material was sixty-two inbred lines from the maize collections belonging to two Polish cultivation companies: The Plant Breeding Smolice IHAR Group (Poland) and the Plant Breeding Małopolska (Poland). Among the analyzed lines were both flint- and dent-shaped grain forms. Lines with a flint-type grain belonged to three different origin groups: F2 (a group related to the F2 line bred at INRA in France from the Lacaune population), EP1 (a group related to the EP1 line, bred in Spain from the population derived from the Pyrenees) and German Flint (a line group bred from the local German population). Lines with a dent-type grain belonged to different origin groups from the United States: Iowa Stiff Stalk Synthetic (BSSS), Iowa Dent (ID) and Lancaster. Inbred lines of complex origin bred from different starting populations and lines of unknown origin were also analyzed (Table 3).

### 4.2. Phenotyping

The field experiment with inbred maize lines was established in 2015 using 10 m^2^ plots in a set of complete random blocks in three replicates in Polish breeding stations in the Plant Breeding Smolice IHAR Group (51°42′20.813″ N, 17°9′57.405″ E) and Plant Breeding Małopolska (50°58′12.75″ N, 16°56′5.892″ E). The analysis of morphological features was conducted from May to October 2015 and included 13 traits: Type of grain (TG), anthocyanin coloration of glumes of cob (ACGC), time of anthesis—50% of flowering plants (TA), time of silk emergence—50% of flowering plants (TSE), anthocyanin coloration of silks (ACSi), anthocyanin coloration of anthers (ACA), anthocyanin coloration at the base of the glume (ACBG), angle between main axis and lateral branches (ANGLE), curvature of lateral branches (CLB), length of main axis above the highest lateral branch (LMA), number of primary lateral branches (NPLB), anthocyanin coloration of sheath (ACSh) and anthocyanin coloration of internodes (ACI). Biometric measurements were carried out in the first half of November 2015 and included nine traits: Plant length (PL), height ratio of insertion of peduncle of the upper ear to plant length (HIP), cob diameter (DC), cob length (LC), number of rows of grain (NRG), number of grains per cob (NGC), weight of fresh grains per cob (WFGC), dry matter content at harvest time (DM) and weight of fresh grains per cob at 15% moisture (WFG15). Measurements concerning yield structure traits were performed on 20 randomly selected cobs from three replicates of each inbred line.

Climatic conditions: In 2015, the average rainfall in Smolice was 39.45 mm and was 5.82 mm lower than the average rainfall for many years. The highest rainfall was in July (55 mm) and the lowest in March (15 mm). The average air temperature this year in Smolice was 11.54 °C and was higher than the average temperature over the years by 1.8 °C. The warmest month in 2015 was August (21 °C), while the lowest temperature was recorded in December (1.1 °C). In 2015, rainfall and temperature levels were unfavorable during the initial development of maize. Despite the early sowing date, the maize remained in the 2–3 leaf stage for a long time, and purple discoloration was visible on the leaves due to the difficulty in taking phosphorus from the soil. May was full of rainfall, which had a positive effect on the further development of maize.

### 4.3. Genotyping and SilicoDArT and SNP Data Processing

Genotype data for association mapping were derived from polymorphisms identified in DArT and candidate gene sequences.

Sixty-two lines were genotyped. Total genomic DNA was extracted from the young leaves of the analyzed forms using the GenElute Plant Mini Kit (Sigma-Aldrich, Poznań, Poland). DNA purity and concentration were determined spectrophotometrically (Thermo Scientific, Waltham, MA, USA). The concentration of all DNA samples was adjusted to 100 ng µL^−1^. The DArTseq analysis was performed at Diversity Arrays Technology Pty Ltd. (Australia).

DNA samples digestion/ligation reactions were processed according to Kilian et al. [13] but replacing a single PstI-compatible adaptor with two adaptors corresponding to: PstI- and NspI-compatible sequences and moving the assay on the sequencing platform as described by Sansaloni et al. [15]. The PstI-compatible adapter was designed to include Illumina flowcell attachment sequence, sequencing primer sequence and “staggered”, varying length barcode region, similar to the sequence reported by Elshire et al. [17]. Reverse adapter contained flowcell attachment region and NspI-compatible overhang sequence.

Only “mixed fragments” (PstI–NspI) were amplified in PCR using the following reaction conditions: Denaturation 1 min at 94 °C, followed by 30 cycles of 94 °C for 20 s, 58 °C for 30 s and 72 °C for 45 s, and the final elongation 72 °C for 7 min. After PCR equimolar amounts of amplification products from each sample of the 96-well microtiter plate are bulked and applied to c-Bot (Illumina) bridge PCR followed by sequencing on Illumina Hiseq2500. The sequencing (single read) was run for 77 cycles.

Sequences generated from each lane were processed using proprietary DArT analytical pipelines. In the primary pipeline the fastq files were first processed to filter away poor quality sequences, applying more stringent selection criteria to the barcode region compared to the rest of the sequence. In that way the assignments of the sequences to specific samples carried in the “barcode split” step were very reliable. Approximately 2,500,000 (+/−7%) sequences per barcode/sample were used in marker calling. Finally, identical sequences were collapsed into “fastqcall files”. These files were used in the secondary pipeline for DArT PL’s proprietary SNP and SilicoDArT (presence/absence of restriction fragments in representation) calling algorithms (DArTsoft14). For the association analysis, only DArT sequences meeting the following criteria were selected: One SilicoDArT and SNP within a given sequence (69 nt), minor allele frequency (MAF) >0.25 and the missing observation fractions <10%. SilicoDArT and SNP sequences were mapped using the Blast service at https://www.gabipd.org/ with default parameters. The sequences (69 bp) of three SilicoDArT markers (4591115, 7059939 and 5587991) were used to search the RefSeq genome database of *Zea mays* (tax ID: 4577) with Nucleotide BLAST search (NCBI, https://blast.ncbi.nlm.nih.gov/Blast.cgi). The silicoDArT markers are dominant, because they represent the presence versus absence of restriction enzyme fragment in genomic representations of a subset of lines in the analysis. These markers are extracted by DArTsoft14 software and markers, which were present in a representation were assigned 1 and those absent were assigned 0 value, respectively.

### 4.4. Statistical Analysis and Association Mapping

A one-way analysis of variance (ANOVA) was performed to verify the hypothesis of the lack of the effect of lines on the variability of observed traits. Sample sizes for lines that were used in calculations were equal to ten for each of the four replications. The coefficients of genetic similarity (S) of the investigated lines were calculated using the Nei and Li [29] formula. Lines were grouped hierarchically using the unweighted pair group method of arithmetic means (UPGMA) based on calculated coefficients. The relationships among lines were presented in the form of a dendrogram. Association mapping was performed using a method based on the mixed linear model with the population structure estimated by the eigenanalysis (principal component analysis applied to all markers) and modeled by random effects [43,44]. All analyses were conducted in Genstat 18.2. Significance of associations between traits and SilicoDArT and SNP markers was assessed on the basis of *p*-values corrected for multiple testing by the Benjamini–Hochberg method.

## 5. Conclusions

The development of new genotyping methods based on hybridization markers or NGS makes them increasingly applied in basic research. The availability of a large number of SNP markers or the reproducibility of DArT technology and their decreasing costs make modern methods to be used in economically important plants in applied research, such as identification of trait markers or even selection at the level of entire genomes, when the criterion of time is more important than the initial financial expenditure. The results of the conducted research show undoubtedly to the advantages of DArT Seq technology because of it identifying 49,911 polymorphisms (including 33,452 SilicoDArT and 16,459 SNP). Of these markers three very important ones were identified, and deserve particular attention, because they were associated with five or six traits: SilicoDArT 4591115, SilicoDArT 7059939 and SilicoDArT 5587991. These markers will be analyzed and tested in subsequent years of research. As results from conducted research molecular markers SilicoDArT and SNP can also be used in this species to group lines in terms of origin and lines with incomplete origin data. They can therefore be used to select parent components for heterosis hybrids. However, one should be aware of the advantages and disadvantages of DArT or SNP markers discussed. It seems that DArT markers may sometimes be more convenient than SNP markers due to their dominant nature (e.g., in polyploid species). The availability of probe DNA sequences, and thus the possibility of developing specific markers is their unquestionable advantage. At the same time, the DArTseq technology (in contrast to GBS) provides a large pool of the so-called silicoDArTs, which are also dominant (such a marker is either present or not in a given genotype, but is not related to the difference in the DNA sequence of a given marker). One should also be aware of the fact that DArT markers, due to their known location in many utility species, may be a better solution than SNP markers when the chromosomal location or localization of linkage groups to specific chromosomes of a given species is important. Therefore, it is worthwhile to consider which type of markers will provide greater advantages in the case of specific research tasks.

## Figures and Tables

**Table 1:** Correlation coefficients of observed traits on the basis of mean values for inbred lines (*n* = 4; m = 10).

Trait	TG	ACGC	TA	TSE	ACSi	ACA	ACBG	ANGLE	CLB	LMA	NPLB	ACSh	ACI	PL	HIP	DC	LC	NRG	NGC	WFGC	DM	WFG15
TG	1																					
ACGC	0.61	1																				
TA	0.38	0.17	1																			
TSE	0.39	0.17	0.79	1																		
ACSi	−0.33	−0.17	−0.31	−0.29	1																	
ACA	−0.16	−0.37	0.04	0.03	0.39	1																
ACBG	−0.14	0.04	−0.07	−0.1	0.31	0.2	1															
ANGLE	−0.13	0.07	−0.21	−0.17	−0.01	−0.09	0.18	1														
CLB	−0.25	−0.32	−0.27	−0.19	−0.04	0.11	0.1	0.51	1													
LMA	0.01	−0.04	0.28	0.22	−0.31	−0.09	−0.17	0.01	0.07	1												
NPLB	0.16	0.11	0.11	0.09	−0.05	0.05	0.29	0.24	−0.1	−0.24	1											
ACSh	−0.37	−0.2	−0.45	−0.4	0.35	0.17	0.28	0.03	0.18	−0.17	0.08	1										
ACI	−0.42	−0.14	−0.42	−0.46	0.48	0.15	0.59	0.25	0.15	−0.35	0.22	0.47	1									
PL	0	−0.26	0.45	0.33	−0.18	0.12	−0.12	−0.26	−0.11	0.31	0.06	−0.27	−0.21	1								
HIP	−0.02	−0.19	0.43	0.2	−0.03	0.24	−0.03	−0.27	−0.17	0.12	0.13	−0.2	−0.03	0.74	1							
DC	0.56	0.28	0.4	0.42	−0.19	0.03	−0.05	−0.09	−0.25	−0.16	0.32	−0.31	−0.34	0.31	0.26	1						
LC	−0.04	−0.09	0.07	0.13	−0.31	−0.29	0.06	0.14	0.25	0.33	−0.08	−0.05	−0.05	0.27	0.04	−0.03	1					
NRG	0.32	0.18	0.17	0.14	−0.07	0.1	−0.17	−0.19	−0.02	−0.05	0.1	−0.15	−0.31	0.2	0.21	0.56	−0.09	1				
NGC	0.32	0.17	0.1	0.12	−0.28	−0.13	−0.06	0.2	0.18	0.08	0.23	−0.22	−0.21	0.17	0.09	0.53	0.5	0.61	1			
WFGC	0.37	0.18	0.27	0.27	−0.23	−0.04	0	−0.09	−0.1	0.1	0.12	−0.17	−0.17	0.39	0.36	0.65	0.49	0.5	0.74	1		
DM	−0.12	−0.16	−0.36	−0.37	−0.11	−0.06	−0.11	0.33	0.26	−0.04	−0.02	−0.08	−0.06	−0.26	−0.34	−0.28	−0.13	−0.24	−0.07	−0.53	1	
WFG15	0.38	0.16	0.2	0.19	−0.27	−0.06	−0.04	−0.02	−0.04	0.08	0.13	−0.22	−0.19	0.37	0.31	0.65	0.5	0.49	0.81	0.97	−0.32	1
	*p* < 0.05	*p* < 0.01	*p* < 0.001																			

TG—type of grain, ACGC—anthocyanin coloration of glumes of cob, TA—time of anthesis (50% of flowering plants), TSE—time of silk emergence (50% of flowering plants), ACSi—anthocyanin coloration of silks, ACA—anthocyanin coloration of anthers, ACBG—anthocyanin coloration at the base of the glume, ANGLE—angle between main axis and lateral branches, CLB—curvature of lateral branches, LMA—length of main axis above the highest lateral branch, NPLB—number of primary lateral branches, ACSh—anthocyanin coloration of sheath, ACI—anthocyanin coloration of internodes, PL—plant length, HIP—height ratio of insertion of the peduncle of the upper ear to plant length, DC—cob diameter, LC—cob length, NRG—number of rows of grain, NGC—number of grains per cob, WFGC—weight of fresh grains per cob, DM—dry matter content at harvest time, WFG15—weight of fresh grains per cob at 15% moisture.

**Table 2:** Associations between markers (Silico and SNP) and studied traits found in genome-wide association mapping with allelic substitution effects (significant associations selected at *p* < 0.05 with correction for multiple testing by the Benjamini–Hochberg method).

Trait Number	Trait	No of Significant Markers			LOD Min			LOD Max			Effect Min			Effect Max		
		Silico	SNP	Total	Silico	SNP	Total	Silico	SNP	Total	Silico	SNP	Total	Silico	SNP	Total
1	Type of grain	40	22	62	2.52	2.53	2.52	7.68	4.62	7.68	−0.99	−0.78	−0.99	0.73	0.82	0.82
2	Anthocyanin coloration of glumes of cob	101	49	150	2.50	2.53	2.50	9.53	10.33	10.33	−1.26	−1.05	−1.26	1.18	1.17	1.18
3	Time of anthesis (50% of flowering plants)	30	29	59	2.54	2.52	2.52	5.35	6.06	6.06	−2.15	−2.64	−2.64	2.33	1.95	2.33
4	Time of silk emergence (50% of flowering plants)	47	24	71	2.51	2.51	2.51	4.99	8.06	8.06	−2.46	−3.22	−3.22	2.53	2.27	2.53
5	Anthocyanin coloration of silks	59	28	87	2.51	2.54	2.51	6.23	5.50	6.23	−1.17	−1.46	−1.46	1.51	1.38	1.51
6	Anthocyanin coloration of anthers	31	8	39	2.50	2.52	2.50	3.67	3.63	3.67	−1.07	−1.08	−1.08	1.29	1.25	1.29
7	Anthocyanin coloration at the base of the glume	38	18	56	2.60	2.53	2.53	5.57	3.63	5.57	−1.30	−1.09	−1.30	1.22	1.11	1.22
8	Angle between main axis and lateral branches	40	17	57	2.51	2.54	2.51	6.78	4.58	6.78	−0.89	−0.82	−0.89	1.12	0.91	1.12
9	Curvature of lateral branches	18	10	28	2.58	2.69	2.58	4.16	4.21	4.21	−0.80	−0.71	−0.80	0.88	0.84	0.88
10	Length of main axis above the highest lateral branch	30	20	50	2.55	2.59	2.55	5.62	4.99	5.62	−0.92	−0.93	−0.93	0.93	0.82	0.93
11	Number of primary lateral branches	5	1	6	2.51	2.98	2.51	3.16	2.98	3.16	0.40	0.44	0.40	0.47	0.44	0.47
12	Anthocyanin coloration of sheath	4	9	13	2.51	2.70	2.51	3.14	4.98	4.98	−0.65	−0.76	−0.76	0.77	0.96	0.96
13	Anthocyanin coloration of internodes	14	6	20	2.56	2.60	2.56	4.05	3.66	4.05	−0.86	−0.70	−0.86	1.11	0.97	1.11
14	Plant length	18	10	28	2.64	2.65	2.64	4.45	4.48	4.48	−16.29	12.55	−16.29	19.27	19.02	19.27
15	Height ratio of peduncle insertion of the upper ear to plant length	26	18	44	2.54	2.62	2.54	5.67	4.82	5.67	−9.25	−8.74	−9.25	10.87	9.76	10.87
16	Cob diameter	16	13	29	2.51	2.54	2.51	4.90	5.29	5.29	−0.18	−0.16	−0.18	0.22	0.19	0.22
17	Cob length	24	13	37	2.51	2.52	2.51	4.70	3.93	4.70	−1.07	−1.22	−1.22	1.63	1.27	1.63
18	Number of rows of grain	16	6	22	2.51	2.54	2.51	4.22	3.66	4.22	−0.91	−0.88	−0.91	0.93	0.81	0.93
19	Number of grains per cob	13	8	21	2.57	2.53	2.53	4.31	3.22	4.31	−34.25	−29.05	−34.25	38.10	32.47	38.10
20	Weight of fresh grains per cob	19	14	33	2.51	2.53	2.51	3.57	5.32	5.32	−15.69	−13.81	−15.69	12.87	18.41	18.41
21	Dry matter content at harvest time	12	8	20	2.52	2.51	2.51	5.20	2.85	5.20	−2.65	−2.13	−2.65	2.92	2.03	2.92
22	Weight of fresh grains per cob at 15% moisture	22	15	37	2.53	2.51	2.51	4.26	4.94	4.94	−9.34	−11.17	−11.17	11.34	13.86	13.86
	Total	623	346	969												

**Table 3:** Plant material used in the experiment with division into origin groups of flint-shaped grain forms, dent-shaped grain forms and semi dent-shaped grain forms inbred lines of maize (*Zea mays* L.).

**Origin Groups of the Lines**	Flint	F2	**Inbred Line Numbers**	74
		F2/EP1		35,39,41,51,53
		F2/CM7		73
		Flint/BSSS		38
		Flint/ID		40
		Flint/Lancaster		60,68
		German Flint/F2		69,82,93
		Origin unknown		42,52,36
	Dent	ID		33,43,44,56,58,59,61,62,64,65,72,76,79,80,83,85,88,89,90,94
		BSSS		47,49,50,87
		ID/BSSS		54,57,63,66,67,75,77,78,81,84,86,91,92,45,46
		Lancaster		55
		ID/Lancaster		50
	Semident	BSSS		71
		Origin unknown		37,34,48

## Supplementary Materials

The following are available online at https://www.mdpi.com/2223-7747/8/9/330/s1, Table S1: Effects of markers (Silico and SNPs) associated with two or more traits (pleiotropy) found in GWAM with allelic substitution effects (significant associations selected at *p* < 0.05 with correction for multiple testing by the Benjamini-Hochberg method).
