# Supplementary material for: Identification of Markers Associated with Yield Traits and Morphological Features in Maize (Zea mays L.)
Source: Plants (Basel). 2019 Sep 5;8(9):330. doi: 10.3390/plants8090330 (PMC6783969; doi:10.3390/plants8090330)
Supplement: Supplementary file 1 [file plants-08-00330-s001.pdf]

Table 1. Effects of markers (Silico and SNPs) associated with two or more traits (pleiotropy) found in GWAM with allelic substitution effects (significant associations selected at P < 0.05 with correction for multiple testing by the Benjamini-Hochberg method).

Marker Type	Marker ID	SNP Position REF>ALT*	Frequency of Alleles		Trait (Effect)																					
			REF	ALT	1	2	3	4	5	6	7	8	9	10	11	12	13	14	15	16	17	18	19	20	21	22
Silic	4582827		0.52	0.48	-0.66																					-8.86
Silic	2462460		0.50	0.50							-1.30						-0.86									
Silic	7048729		0.67	0.33		-1.09						-0.79														
Silic	2567297		0.67	0.33																				10.58		8.53
Silic	2507245		0.52	0.48			2.33	2.53											7.22							
Silic	9682161		0.55	0.45			2.17	2.43										14.38	7.82							
Silic	2496310		0.72	0.28															-8.48					-11.19		-9.24
Silic	5589517		0.63	0.38		-0.84				1.06																
Silic	2484574		0.71	0.29	0.57	0.88																				
Silic	2463605		0.56	0.44																				11.19		9.46
Silic	4581341		0.69	0.31																				-12.24		-9.34
Silic	2623186		0.52	0.48										0.69				14.74								
Silic	2511480		0.66	0.34																				-11.13		-8.69
Silic	9708613		0.64	0.36					-0.98																	8.68
Silic	4764314		0.52	0.48																				-10.68		-8.87
Silic	4774463		0.63	0.37			1.92	1.93						0.73												
Silic	7057245		0.68	0.32									-0.71										-28.32			
Silic	100000291		0.65	0.35	-0.99	-0.95																				
Silic	100000311		0.72	0.28														18.24			1.06					
Silic	4767079		0.61	0.39	0.62	0.91																				
Silic	100000332		0.54	0.46				-1.83											8.72							
Silic	4591397		0.63	0.38					0.91			-0.70														
Silic	2383289		0.71	0.29	0.58	0.75																				
Silic	9693871		0.58	0.42	0.70			-2.18																		
Silic	4579407		0.72	0.28										0.72				18.38								
Silic	4591115		0.50	0.50					-1.07					0.80							0.87		31.59	10.74		9.59
Silic	4579950		0.72	0.28	-0.66	-0.85																				
Silic	2604422		0.64	0.36																			26.97			9.40
Silic	4577424		0.54	0.46	0.52	0.75																				
Silic	7059931		0.52	0.48			-2.10				-0.90															
Silic	7053630		0.71	0.29		-1.11		-2.31	1.20																	
Silic	4586610		0.60	0.40		-0.81																		-9.89		-7.98
Silic	4576907		0.69	0.31								0.70	0.73													
Silic	4765803		0.73	0.27									0.73								1.01					

Silic	4577023	0.66	0.34	-0.60			-2.37										-0.15						
Silic	9642120	0.64	0.36																		12.50		10.34
Silic	4768225	0.71	0.29								0.74		0.47										
Silic	4770201	0.63	0.37																		11.70		9.11
Silic	4778141	0.61	0.39	-0.51								-0.72											
Silic	21695474	0.56	0.44			1.75										7.88							
Silic	4775155	0.54	0.46							0.97	0.81												
Silic	4588209	0.53	0.47							0.93									-0.72				
Silic	7059939	0.54	0.46		-0.88	-1.65	-1.86	0.99									-0.16						
Silic	4578562	0.58	0.42		-0.75		-1.82		1.02														
Silic	4778705	0.53	0.47		-0.71				0.98														
Silic	4765436	0.52	0.48						0.91							7.96							
Silic	4576389	0.67	0.33								0.82											2.92	
Silic	7061446	0.61	0.39						1.02							8.44							
Silic	4769484	0.58	0.42		-0.75		-1.87		1.02														
Silic	21698094	0.57	0.43					1.01										-0.96		-34.25			-8.50
Silic	4589183	0.61	0.39					0.94										-0.92		-31.99			-8.65
Silic	16720800	0.72	0.28												19.27			1.22					
Silic	4584774	0.70	0.30					1.08		1.10													
Silic	4590177	0.71	0.29							1.10		-0.92											
Silic	7054794	0.57	0.43						1.01									-0.82					
Silic	4776633	0.68	0.32					1.29		1.05													
Silic	4580026	0.70	0.30			-1.99	-2.46	1.15				-0.82											
Silic	4590817	0.71	0.29	-0.82	-0.79			1.07											-0.80				
Silic	4589725	0.70	0.30					1.16		1.03													
Silic	4593946	0.58	0.42						0.90							7.13							
Silic	4579295	0.64	0.36									-0.71		0.76									
Silic	4588727	0.59	0.41									0.69			16.65								
Silic	9686211	0.53	0.47																		10.77		8.97
Silic	100000598	0.72	0.28							1.08	0.98									34.89			
Silic	4775187	0.52	0.48																		-10.98		-8.62
Silic	16720367	0.70	0.30		-0.84										15.49								
Silic	2447022	0.60	0.40				-2.17			-1.04									0.70				
Silic	4588356	0.50	0.50									0.78				7.17							
Silic	2536224	0.66	0.34	0.51	0.77			-1.03															
Silic	4778846	0.71	0.29				1.66				0.71												
Silic	100001068	0.60	0.40														-0.15				-10.92		-9.11
Silic	7047417	0.55	0.45												-14.66	-8.25							
Silic	2376603	0.54	0.46								-0.77	0.83											
Silic	4583458	0.69	0.31																	35.26	12.87		11.34
Silic	4778577	0.57	0.43		-1.04		1.88																

Silic	4587600		0.52	0.48		0.74	1.97	1.65	-1.01																
Silic	4767781		0.55	0.45								0.73								0.85					
Silic	4588313		0.64	0.36	0.50	0.80																			
Silic	4580567		0.61	0.39		0.64							-0.72						0.15						
Silic	4778526		0.66	0.34			1.61	1.69																	
Silic	4778001		0.71	0.29			1.68	1.93																-2.26	
Silic	100001978		0.71	0.29					1.39											-1.07					
Silic	2490401		0.70	0.30										-0.81	0.44										
Silic	4591628		0.64	0.36							1.06	0.84													
Silic	4775023		0.57	0.43														7.49			0.78				
Silic	4768116		0.72	0.28			1.70	1.70																	
Silic	4768235		0.72	0.28			1.75						-0.77												
Silic	9625831		0.70	0.30																			12.20	-2.01	
Silic	4766961		0.70	0.30																	0.92	38.10	12.40		
Silic	4575873		0.54	0.46		-0.82				0.95															
Silic	4765788		0.71	0.29		-0.88											16.60								
Silic	7054550		0.70	0.30	0.63				-1.06																
Silic	4576700		0.63	0.38	0.47				-1.13																
Silic	4579180		0.71	0.29				-2.44	1.51																
Silic	9631643		0.63	0.38		-0.81							0.70						-0.16						
Silic	100002443		0.70	0.30							1.17					1.11									
Silic	4585140		0.72	0.28				-2.33											-0.15		-0.83				
Silic	4586501		0.71	0.29	-0.73	-0.83			1.41																
Silic	9627047		0.67	0.33	0.70														0.22			30.40			11.32
Silic	7053980		0.73	0.27		-0.79							0.88												
Silic	4577791		0.69	0.31		-0.74							0.75												
Silic	9677395		0.71	0.29	-0.71	-0.92																			
Silic	9683236		0.70	0.30	-0.74	-1.00		-2.35	1.22																
Silic	100002879		0.72	0.28					1.12		1.07														
Silic	100002907		0.72	0.28									0.72						-0.17						
Silic	5587991		0.73	0.27		-0.82	-2.07		1.03				0.74								-0.87				
Silic	2518294		0.71	0.29	-0.83	-1.26							0.72												
Silic	100003085		0.71	0.29	0.68	0.99																			
Silic	4588359		0.66	0.34	0.73	1.07																			
SNP	4771733	51: C>A	0.58	0.42					0.94									-8.17							
SNP	4771414	54: A>G	0.55	0.45																			10.97		9.46
SNP	4772623	57: T>C	0.58	0.42		0.68	1.57																		
SNP	7057079	7: T>C	0.56	0.44		0.73	1.62	1.66																	
SNP	4771759	49: G>A	0.64	0.36							0.95			-0.70											
SNP	2482627	67: C>G	0.61	0.39					-0.96					0.73											
SNP	4775006	17: A>C	0.53	0.47	0.82	0.84			-1.46																

SNP	2457095	12: G>T	0.51	0.49			1.84									14.69	9.13						
SNP	2429301	62: G>C	0.58	0.42	0.53	0.77	1.72																
SNP	4582630	40: G>A	0.69	0.31							1.09			-0.82	0.93								
SNP	2435775	32: C>T	0.53	0.47																	18.41		13.86
SNP	4582418	47: A>G	0.59	0.41																	-11.46		-8.51
SNP	2526788	25: C>A	0.73	0.27												19.02			1.27				
SNP	4771078	54: G>A	0.63	0.37					1.29			-0.75											
SNP	2379065	43: T>C	0.51	0.49										0.82					0.91				
SNP	2426266	50: T>C	0.54	0.46												14.83	8.76						
SNP	4583437	30: G>C	0.56	0.44					1.13					-0.74									
SNP	4593266	24: A>G	0.72	0.28			-2.64	-2.98					0.73										
SNP	9710852	60: T>C	0.70	0.30			-2.56	-3.22	1.24									-0.16					
SNP	2395244	7: C>G	0.60	0.40	0.49	0.92																	
SNP	4576631	22: G>C	0.59	0.41	0.49	1.95	1.67																
SNP	4770164	18: A>G	0.71	0.29		-1.05		-2.33															
SNP	2509909	57: C>T	0.52	0.48								0.77	0.84										
SNP	4772780	19: T>C	0.59	0.41			1.89	1.89						0.69									
SNP	4593510	13: G>T	0.66	0.34	0.50	0.84																	
SNP	4593789	12: G>T	0.57	0.43								-0.82					8.86						
SNP	4590061	61: C>G	0.66	0.34					1.15					-0.78									
SNP	4575906	27: A>C	0.56	0.44					-0.94					0.65									
SNP	4584241	61: A>G	0.71	0.29				1.87										0.19					
SNP	2429645	31: G>A	0.64	0.36		0.70						0.74											
SNP	4766586	35: C>A	0.69	0.31	0.60		1.85											0.19					
SNP	4765276	17: G>C	0.61	0.39			1.70	2.03						0.67					0.89				
SNP	4577076	30: C>A	0.51	0.49															-0.86				-8.20
SNP	4772822	33: T>G	0.56	0.44																	10.27		8.67
SNP	4581735	7: A>T	0.56	0.44	-0.49	-0.66																	
SNP	4772346	5: C>T	0.53	0.47			1.51										8.11	0.15					
SNP	4768034	20: T>G	0.54	0.46			1.42	2.27															
SNP	7061083	10: T>C	0.58	0.42																	13.31		11.17
SNP	9704516	20: C>A	0.52	0.48			-1.87	-1.54		1.10													
SNP	4582139	29: G>A	0.61	0.39			1.81												-0.94				
SNP	4764143	27: G>A	0.53	0.47																	12.17		9.19
SNP	4772190	18: A>G	0.58	0.42																	-10.53	2.03	
SNP	100027248	7: A>G	0.51	0.49		0.86				-1.08													
SNP	4591311	8: A>T	0.69	0.31					1.01					-0.83									
SNP	4777014	14: C>T	0.55	0.45														-0.14		-0.71			
SNP	4576829	13: G>A	0.61	0.39			1.62	2.17															
SNP	4770814	28: T>G	0.61	0.39	0.59													0.16					
SNP	2391444	26: T>C	0.60	0.40			-1.58														-10.35		

SNP	4591953	38: A>T	0.70	0.30				-2.60	1.22		1.00													
SNP	4772456	27: A>T	0.66	0.34							0.95									-0.88				
SNP	2393968	55: T>C	0.63	0.38																	-26.71			-9.04
SNP	4771373	10: T>A	0.52	0.48		-0.64								-0.63										
SNP	2528450	39: C>T	0.54	0.46																		-12.33		-9.34
SNP	4583777	47: A>G	0.56	0.44					0.98	1.09														
SNP	7060941	7: A>C	0.50	0.50			1.45	1.91																
SNP	4581940	65: T>C	0.59	0.41					1.14					-0.87										
SNP	9716833	19: C>T	0.68	0.32								0.86	0.84											
SNP	2393771	29: C>T	0.52	0.48			-1.78		0.90															
SNP	4583665	9: C>T	0.55	0.45														0.14					-2.13	
SNP	21697847	15: G>C	0.70	0.30	-0.56																	-13.81		-11.17
SNP	21699058	5: A>G	0.54	0.46					1.16					-0.89					-0.94					
SNP	7048563	18: A>C	0.53	0.47																		10.85	-1.98	
SNP	4582235	26: A>C	0.55	0.45		-0.68											8.57							
SNP	4584576	40: G>C	0.54	0.46												13.63					32.47	12.81		11.58
SNP	4592288	43: T>C	0.60	0.40			-2.15								0.68							`		
SNP	7061574	45: C>T	0.64	0.36	0.62													0.17						
SNP	2589761	13: C>T	0.66	0.34																		-12.03		-8.94
SNP	4778235	25: G>A	0.53	0.47																	28.36			9.11
SNP	5587157	15: T>C	0.50	0.50				1.79		-1.01														
SNP	7050191	16: A>G	0.56	0.44			-2.53	-1.89																
SNP	7061258	28: G>T	0.59	0.41			1.92	1.93						0.66										
SNP	4764785	17: T>C	0.63	0.37	-0.52	-0.91				0.95		-0.73												
